# Conversation between skin microbiota and the host: from early life to adulthood

**DOI:** 10.1038/s12276-025-01427-y

**Published:** 2025-04-01

**Authors:** Jimin Cha, Tae-Gyun Kim, Ji-Hwan Ryu

**Affiliations:** 1 Department of Biomedical Sciences, Yonsei University College of Medicine, Seoul, Republic of Korea; 2 Brain Korea 21 Project, Yonsei University College of Medicine, Seoul, Republic of Korea; 3 Department of Dermatology, Severance Hospital, Cutaneous Biology Research Institute, Yonsei University College of Medicine, Seoul, Republic of Korea; 4 Institute for Immunology and Immunological Diseases, Yonsei University College of Medicine, Seoul, Republic of Korea

**Keywords:** Immunology, Molecular biology

## Abstract

Host life is inextricably linked to commensal microbiota, which play a crucial role in maintaining homeostasis and immune activation. A diverse array of commensal microbiota on the skin interacts with the host, influencing the skin physiology in various ways. Early-life exposure to commensal microbiota has long-lasting effects, and disruption of the epidermal barrier or transient exposure to these microorganisms can lead to skin dysbiosis and inflammation. Several commensal skin microbiota have the potential to function as either commensals or pathogens, both influencing and being influenced by the pathogenesis of skin inflammatory diseases. Here we explore the impact of various commensal skin microbiota on the host and elucidate the interactions between skin microbiota and host systems. A deeper understanding of these interactions may open new avenues for developing effective strategies to address skin diseases.

## Commensal microbiota shape skin health and immune response

The skin is our body’s largest organ and acts as a barrier against the outside world. It hosts a variety of tiny organisms, known as microbiota, which include bacteria, fungi and viruses. These microbiota help to keep our skin healthy. However, scientists are still learning how these organisms interact with our skin and immune system. Researchers have found that certain bacteria, such as *Staphylococcus epidermidis*, help to repair skin and maintain its health. They conducted studies using mice and human skin samples to understand these interactions better. The research showed that these bacteria can influence immune responses and even help in healing wounds. The study highlights that the balance of skin microbiota is crucial for preventing skin diseases such as atopic dermatitis and psoriasis. When this balance is disrupted, it can lead to inflammation and other skin issues.

This summary was initially drafted using artificial intelligence, then revised and fact-checked by the author.

## Introduction

The skin serves as the primary epithelial barrier, separating the internal body from the exterior environment and interacting with various environmental factors. The skin contains a set of specialized structures of appendages, such as hair follicles, sweat glands and sebaceous glands, which support physiological homeostasis. These provide nutrients and form areas that make the skin a conducive environment for commensal microbiota to thrive^1,2^. A diverse range of commensal microbiota, such as bacteria, fungi, viruses, microeukaryotes, archaea and phages, interact with host skin through a variety of mechanisms^3^.

Commensal skin microbiota contribute to maintaining homeostasis by stimulating the skin. For example, *Staphylococcus epidermidis* not only plays a role in maintaining the homeostasis of host skin but also promotes wound repair^4–10^. *Staphylococcus lugdunensis* reduces inflammation and maintains host skin homeostasis by inhibiting the growth of *Staphylococcus aureus*. In addition, *Staphylococcus hominis* exhibits antimicrobial activity that inhibits *S. aureus* growth, thereby sustaining host skin homeostasis^11,12^.

The skin senses touch, pain, itching and temperature changes via sensory neurons^13,14^. Moreover, some commensal microbiota are associated with the stimulation of these sensory neurons. *S. aureus* promotes the regeneration of peripheral sensory neurons upon injury and induces itching and tissue damage by activating proteinase-activated receptor 1 (PAR1) in neurons through protease secretion^15,16^. Furthermore, sensory neurons in the skin induce IL-17A responses upon recognizing *Candida albicans*^17^, while α-hemolysin derived from *S. aureus* and streptolysin S derived from *Streptococcus pyogenes* elicit pain by stimulating neurons^18,19^. Moreover, Toll-like receptor (TLR) 4 recognizes lipopolysaccharides from Gram-negative bacteria, inducing itching via histamine^20,21^.

In addition, some commensal microbiota influence the regulation of skin immune cells. For instance, *S. epidermidis* affects the type 17 response through skin dendritic cell (DC) interaction, regulatory T (T_reg_) cell recruitment and mucosal-associated invariant T cell imprinting^5–10,22^. Moreover, indole-3-aldehyde (IAld)-producing microbiota, such as *Staphylococcus lentus*, prime skin group 2 innate lymphoid cells (ILC2s) during the specific postnatal period^23^. Some commensal skin microbiota promote RORδt^+^ IL-17A-producing innate lymphoid cells (ILCs)^24^. Furthermore, skin immune cells actively regulate the skin microbiota population; for example, the depletion of skin ILCs leads to the enlargement of sebaceous glands, which, in turn, induces skin dysbiosis^25^.

The upper portion of hair follicles provides a key habitat for commensal skin microbiota, which induces hypoxia-inducible factor 1 subunit α (HIF-1α) signaling and glutamine metabolism in keratinocytes, promoting hair follicle regeneration^26^. Furthermore, commensal skin microbiota residing in hair follicles augment T_reg_ cell migration^27^. The scalp microbiota of patients with alopecia areata differ from those of healthy controls^28^, suggesting that commensal skin microbiota are associated with hair cycle regulation. Therefore, skin microbiota are involved in many aspects of skin physiology and pathology.

## Commensal skin microbiota and host immune system

### Interactions between commensal skin microbiota and skin epithelial cells

The skin comprises three main layers: the epidermis, dermis and subcutaneous tissue. The epidermis is the outermost layer of the skin and is directly exposed to the external environment. Therefore, diverse commensal microbiota reside in the surface of epidermis^29^. This environment fosters interactions between commensal skin microbiota and the epidermis.

**Fig. 1:**
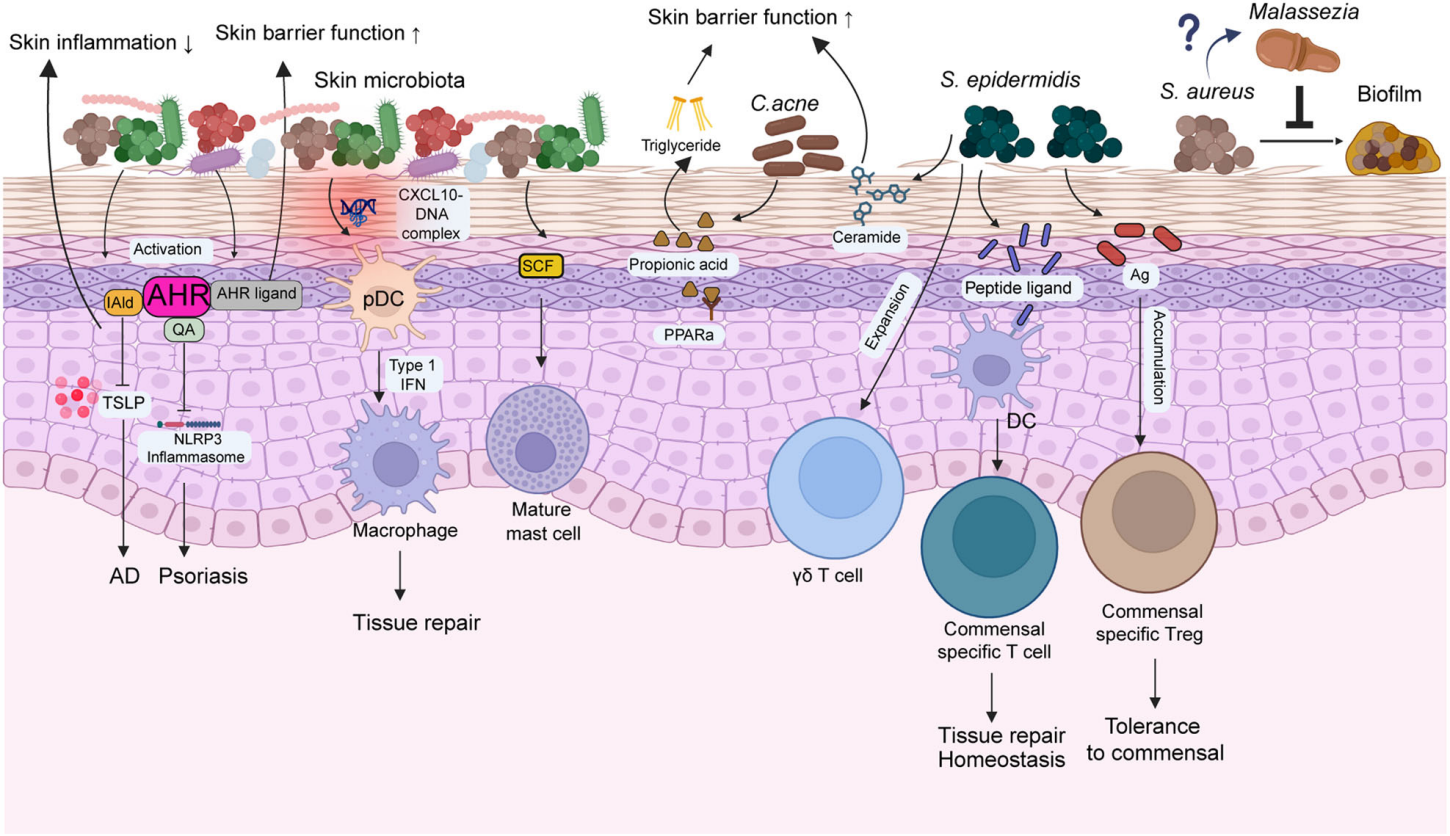
Various roles of skin microbiota. Skin microbiota regulate both skin homeostasis and barrier function. They enhance barrier function by activating the AhR pathway in keratinocytes. In addition, metabolites (IAld and quinolinic acid) from skin microbiota relieve skin inflammation by activating AHR signaling in keratinocytes. This pathway inhibits TSLP and the NLRP3 inflammasome, thereby attenuating atopic dermatitis and psoriasis. Commensal microbiota colonization of skin wounds shape CXCL10–bacterial DNA complexes, which activate plasmacytoid dendritic cells (pDCs) to produce type I interferons. These pDCs promote tissue repair through macrophage-mediated processes. Commensal skin microbiota stimulate keratinocytes to produce stem cell factors (SCFs), which induce mast cell maturation. *S. epidermidis* strengthens the skin barrier, promotes tissue repair, maintains homeostasis and induces tolerance to commensal microorganisms. This is achieved by producing ceramides and inducing commensal-specific T cells through interactions with DCs and T_reg_ cells via peptide ligand and antigen recognition. In addition, *S. epidermidis* can exacerbate skin inflammation through the expansion of γδ T cells. *C. acnes* also supports skin barrier function by producing triglycerides and can similarly contribute to inflammation via γδ T cell expansion. *Malassezia*, a skin fungus, inhibits biofilm formation of *S. aureus*.

The epidermis is crucial in protecting against infections and preventing water loss^29^. Commensal microbiota residing in the epidermis contribute to host skin function as well. Commensal skin microbiota reduce skin water loss by adjusting aryl hydrocarbon receptor (AhR) signaling and enhance the skin barrier by increasing skin repair functions^30^ (Fig. 1). *S. epidermidis* reinforces skin barrier homeostasis by producing sphingomyelinase, which generates ceramides^4^ (Fig. 1). *Cutibacterium acnes* activates peroxisome proliferator-activated receptor-α (PPARα) in host skin through propionic acid production, which induces triglyceride generation and strengthens skin barrier function^31^ (Fig. 1). In addition, specific microbiota, such as *Lactobacillus rhamnosus GG* and *Bifidobacterium longum*, enhance the tight junction function of human keratinocytes^32^. Thus, various commensal skin microbiota directly influence epidermal barrier function. Moreover, keratinocytes use various mechanisms to recognize and interact with commensal skin microbiota.

Keratinocytes recognize commensal microbiota, such as *S. epidermidis* and *C. acnes*, via TLRs, which initiate an innate immune response^33,34^. Moreover, keratinocytes perceive *S. aureus* through pattern recognition receptors, such as TLR1, TLR2, TLR6, nucleotide-binding oligomerization domain 2 and peptidoglycan recognition proteins 3 and 4 (ref. ^35^). In addition, the association of commensal microbiota with mouse skin activates keratinocytes, leading to the upregulation of major histocompatibility complex class II expression, enhanced antigen recognition and the provocation of an immune response^36^. Tryptophan metabolites derived from commensal skin microbiota alleviate inflammation in keratinocytes via AhR signaling^37^ (Fig. 1). Inhibiting the microbial metabolite recognition pathway in keratinocytes may regulate skin inflammation^37^. Quinolinic acid, a metabolite derived from skin microbiota, relieves psoriatic inflammation by inhibiting the NLRP3 inflammasome in keratinocytes via AhR signaling^38^ (Fig. 1). Therefore, keratinocytes have various pathways through which they recognize microorganisms. As microorganisms directly reside on keratinocytes, other molecular mechanisms of microorganism recognition by keratinocytes remain to be discovered. By elucidating the pathways by which specific microbiota influence skin inflammation and appropriately inhibit these pathways, modulating microbiota to relieve skin inflammation may be possible.

The epidermis is critical to maintaining homeostasis by strengthening the epidermal barrier, which serves as the primary defense against external threats. Therefore, a deficiency in genes associated with the skin epidermis not only leads to spontaneous inflammation but also alters the composition of the commensal skin microbiota.

Filaggrin preserves skin barrier function in the stratum corneum, the outer layer of the epidermis. Filaggrin-deficient mice exhibit spontaneous inflammation and an increased abundance of *Staphylococcus* species in the skin microbiota compared with that of wild-type mice^39^ (Table 1). In addition, the type 17 immune response is augmented in filaggrin-deficient mice^40^. Filaggrin deficiency in humans is associated with atopic dermatitis and leads to changes in skin microbiota composition relative to that in healthy controls^41–43^ (Table 1).

**Table 1:** Impact of gene deficiency on skin microbiota composition.

Epidermis–skin microbiota interaction			
Deficient gene	Alteration of skin microbiota	Function	Reference
Filaggrin	Mouse: *Staphylococcus* ↑ Human: *Staphylococcus caprae* ↑ *Finegoldia* ↓ *Anaerococcus* ↓ *Peptoniphilus* ↓	Filaggrin preserves skin barrier function in the stratum corneum, the outer layer of the epidermis. Filaggrin mutant mice exhibit spontaneous dermatitis. Filaggrin deficiency in humans is associated with atopic dermatitis	^39,42,43^
*Tmem79*	Mouse: Overall bacterial load ↑ *Staphylococcus aureus* ↑	*Tmem79* is expressed in the stratum granulosum cells of the epidermis and strengthens skin barrier function. *TMEM79-*mutation in humans shows a weak but considerable effect. *Tmem79*-deficient mice exhibit spontaneous dermatitis.	^45,46^
*Adam17*	Mouse: *Corynebacterium mastitidis* ↑ *Staphylococcus aureus* ↑ *Corynebacterium bovis* ↑	Maintains the skin barrier by regulating EGFR ligands. ADAM17 depletion in the mouse epidermis leads to atopic dermatitis development. An SNP in ADAM17 is associated with the allergic march in the Korean population.	^48,49^
Serine protease inhibitor Kazal-type 5 gene (*SPINK5*)	Human: *Staphylococcus* ↑ *Corynebacterium* ↑ Proteobacteria ↓ *Enhydrobacter* ↓ Bacteroidetes ↓ Ascomycota ↑	*SPINK5* gene encodes a protein, LEKT1, a serine peptidase inhibitor. Netherton syndrome, caused by null mutations in *SPINK5*, is marked by widespread erythroderma with scaling and atopic features.	^132^
JunB	Mouse: Total bacterial load ↑ *Staphylococcus aureus* ↑	Epithelial-cell-specific depletion of JunB in mice causes severe skin inflammation, characterized by hyperkeratosis and immune cell infiltration.	^133^
*Suppressor of tumorigenicity 14* (*St14*)	Mouse: *Pseudomonas* ↓ *Corynebacterium* ↑ *Streptococcus* ↑	Matriptase, a serine protease encoded by *St14*, regulates the in vivo processing of profilaggrin to filaggrin. *St14*-deficient mice display acanthosis and orthohyperkeratosis—features common to ichthyotic disorders. In humans, mutations in the *ST14* locus are responsible for autosomal recessive ichthyosis with hypotrichosis.	^134^
G-protein-coupled receptor 15 (*Gpr15*)	Mouse: Bacteriodetes ↑	*Gpr15* acts as a co-receptor for the entry of human immunodeficiency and simian immunodeficiency viruses, activated by the peptide AP57. *Gpr15* deficiency notably alters the composition of skin-resident T cell populations.	^135^
*Adam10*	Mouse: *Corynebacterium mastitidis* ↑	*Adam10* has been implicated in regulating key signaling pathways involved in skin morphogenesis and homeostasis. In mice with *Adam10* ablation from uHF cells, long-term observation following poly(I:C) injection revealed the development of progressive alopecia. These mice also displayed reduced hair pigmentation and lymphocytic infiltration in hair follicles by day 10, which preceded the onset of alopecia.	^64^
Immune function–skin microbiota interaction			
Deficient gene	Alteration of microbiota	Function	Reference
*RAG2*	Mouse: Planococcacea ↑ Staphylococcacea ↑ Human: *Cutibacterium acnes* ↓ *Escherichia coli* ↓ *Corynebacterium pseudogenitalium* ↓	*Rag2*-deficient mice exhibit low adaptive immunity. Null mutations in the *RAG* genes lead to severe combined immune deficiency in individuals, resulting in a deficiency of T and B cells and increased vulnerability to severe infections. Hypomorphic *RAG* mutations cause a mix of immunodeficiency and immune dysregulation in humans, affecting both central and peripheral tolerance, and can present a wide range of clinical symptoms, from recurrent severe infections to autoimmune conditions.	^25,65^
*Rag2* and *Il2rg*	Mouse: Bacteroidales *↑* Bacilli *↓*	Mice deficient in both *Rag2* and *Il2rg* show deficiencies in innate immunity.	^25^
*Il4ra*	Mouse: Firmicutes ↑ Bacteroidetes ↓ Human: *Staphylococcus* ↓	*Il4ra*-deficient mice at a SPF facility developed prominent facial folds and blepharitis, along with loss of coat pigmentation, acanthosis, abnormal hair follicle morphology and enlarged sebaceous glands. In humans, IL-4Rα blockade improves moderate-to-severe atopic dermatitis manifestations and symptoms.	^63,136^
*Nfkbiz*	Mouse: *Pseudomonas* ↓ *Acinetobacter* ↓ *Gemella* ↓ *Ochrobactrum* ↓ *Rhodococcus* ↓ *Staphylococcus xylosus* ↑	IκBζ, encoded by the *Nfkbiz* gene, is a member of the nuclear IκB protein family that functions as a transcriptional regulator through its association with NF-κB. Beginning around 4 weeks of age, *Nfkbiz*-deficient mice exhibited erosion and hair loss in the ocular region, which then extended to the entire body.	^137^
Caspase14	Mouse: Staphylococcaceae ↓ *Stenotrophomonas* ↑ *Acinetobacter* ↑ *Caulobacter* ↑ Oxalobacteriaceae ↑ Enterobacteriaceae ↑ Comamondaceae ↑ Methylobacteriaceae ↑ Enterococcaceae ↑ Paenibacillaceae ↑ Pseudomonadaceae ↑	Caspase-14, an important protease involved in filaggrin catabolism, is primarily functional in fully differentiating keratinocytes, where it is essential for producing natural moisturizing factors in the skin.	^138^
IL-13	Human: *Staphylococcus aureus* ↓	In humans, the inhibition of IL-13 alleviates inflammation and clinical disease activity in atopic dermatitis.	^139^
*Nod2*	Mouse: *Pseudomonas aeruginosa* ↑ *Staphylococcus epidermidis* ↓	NOD2 is an intracellular receptor that detects the muramyl dipeptide motif derived from bacterial peptidoglycans found in bacteria. The skin of *Nod2*-deficient mice is similar to that of wild-type mice.	^140^

*SPF* specific pathogen free, *uHF* upper hair follicle.

Transmembrane protein 79 (TMEM79) is expressed in the stratum granulosum keratinocytes of the epidermis and strengthens skin barrier function^44^. *Tmem79*-deficient mice exhibit spontaneous dermatitis and alterations in the abundance and composition of skin microbiota^45^ (Table 1). Indeed, *TMEM79* mutation in humans shows a weak but important effect^46^.

A disintegrin and metalloprotease 17 (ADAM17) maintains the skin barrier by regulating epidermal growth factor receptor ligands^47^. Depletion of *Adam17* in the mouse epidermis leads to impaired epidermal growth factor receptor signaling, resulting in skin microbiota dysbiosis and the development of atopic dermatitis^48^ (Table 1). In the Korean population, a single-nucleotide polymorphism in *ADAM17* has been associated with the allergic march^49^.

Taken together, deficiency in genes related to the epidermal barrier leads to alterations in skin microbiota and skin inflammation (Table 1). Therefore, the epidermal barrier and the balance of skin microbiota play a pivotal role in maintaining homeostasis.

### Interactions between commensal skin microbiota and skin immune cells

Skin-resident immune cells, such as macrophages, mast cells, DCs, γδ T cells and ILCs, are closely related to commensal skin microbiota^50–52^. Skin-resident macrophages regulate deep skin bacterial infections by controlling the balanced hyaluronic acid degradation^53^. In addition, when a wound occurs in the skin, commensal skin microbiota promote wound repair via the type 1 interferon response through stimulating resident macrophages^54^ (Fig. 1).

Mast cell maturation is induced by stem cell factors derived from keratinocytes influenced by commensal skin microbiota^55^ (Fig. 1). Dermal fibroblasts mediate the development of mast cell tolerance to commensal skin microbiota^56^. In addition, δ-toxin induces mast cell degranulation, leading to inflammation and itching^57^. While *Staphylococcus* δ-toxin is known to activate mast cells, evidence linking skin-microbiota-mediated mast cell activation to itching remains limited^58^. Moreover, studies on the interaction between skin microbiota alterations and mast cell function are insufficient. Elucidating the crosstalk among skin microbiota, mast cells and itching could offer critical insights into skin health and disease.

Skin-resident DCs recognize numerous commensal microbiota and relay this information to other immune cells, thereby supporting wound repair and maintaining homeostasis^5,9,54,59^. Dermal γδ T cells, mainly located at the skin barrier, proliferate in response to *Corynebacterium* and *S. epidermidis*, contributing to skin inflammation^60–62^ (Fig. 1). ILC2s, a subset of ILCs, reside in the skin and are activated by tryptophan-metabolite-producing *Staphylococcus*, thereby modulating adult skin inflammation^23^. In the skin, colonization by *Demodex* induces the expansion of activated ILC2s, which play a critical role in constraining *Demodex* accumulation^63^. CCR6^+^ RORγt^+^ ILCs regulate the sebaceous glands, which, in turn, control the balance of commensal skin microbiota^25^. Skin microorganism dysbiosis induced by epidermal barrier disruption prompts inflammation through ILC2s^64^. In addition, skin microbiota facilitate RORγt^+^ IL-17A-producing ILCs in the early stage of *Leishmania major* infection, thereby exacerbating skin inflammation^24^.

Skin immune cells perceive skin microbiota through multiple pathways. DCs in mouse skin detect *S. epidermidis*-derived peptide ligands, leading to the accumulation of commensal-specific T cells^9^ (Fig. 1). In addition, skin CD11b^+^ conventional DC2s recognize antigens from *S. epidermidis* and *S. aureus*, thereby stimulating antigen-specific T cells^59^. Moreover, neutrophils recognize commensal skin microbiota via TLR2 in response to skin injury^54^.

Furthermore, the association of skin microbiota in the host skin leads to the activation or development of various other immune cells. The activation of *S. epidermidis*-specific CD4^+^ and CD8^+^ T cells during skin colonization by *S. epidermidis* is associated with skin homeostasis and tissue repair^10^, and *S. epidermidis* skin colonization promotes the accumulation of skin T_reg_ cells, consequently preserving skin homeostasis^6^ (Fig. 1). Moreover, exposure to riboflavin-producing commensals primes skin-mucosal-associated invariant T cells, leading to long-lasting effects promoting tissue repair^22^.

Several skin immune cells are influenced by commensal skin microbiota, and in turn, these immune cells affect skin microbiota composition. Mice deficient in recombination-activating gene 2 exhibit a deficiency in adaptive immunity, while those deficient in both recombination-activating gene 2 and interleukin-2 receptor subunit gamma show additional deficiencies in innate immunity. The composition of commensal skin microbiota in these mice is altered compared with that in wild-type mice^25^ (Table 1). Likewise, in humans, Rag deficiency induces alterations in skin microbial diversity relative to that in wild-type gene holders^65^. Alterations in skin microbiota occur when the immune system is disrupted, indicating that skin microorganisms not only influence immune cells but also interact closely with them, suggesting a bidirectional relationship.

### Crosstalk between microbiota and epithelial and immune cells

Commensal skin microbiota inhabit the epidermis, thereby directly affecting the epidermis and modulating its response. This interaction coordinates skin immunity and influences the host fitness. ADAM10 deficiency in type 1 interferon-responsive hair follicle cells in the epidermis inhibits β-defensin-6 in Notch signaling, leading to skin microbiota imbalance and the expansion of *Corynebacterium mastitidis* (Table 1). Consequently, the dysbiosis induces ILC2-mediated skin inflammation and permanent hair loss^64^. Furthermore, colonization of the skin by *Staphylococcus* species capable of producing the tryptophan metabolite IAld leads to increased thymic stromal lymphopoietin (TSLP) expression in epidermal keratinocytes through the action of IAld. This, in turn, primes skin ILC2s to produce IL-5, which exacerbates skin inflammation^23^. Thus, the interaction between microbiota and the host involves notable crosstalk between microbiota and epithelial and immune cells.

## Imprinting of the skin immune system by commensal microbiota in early life

**Fig. 2:**
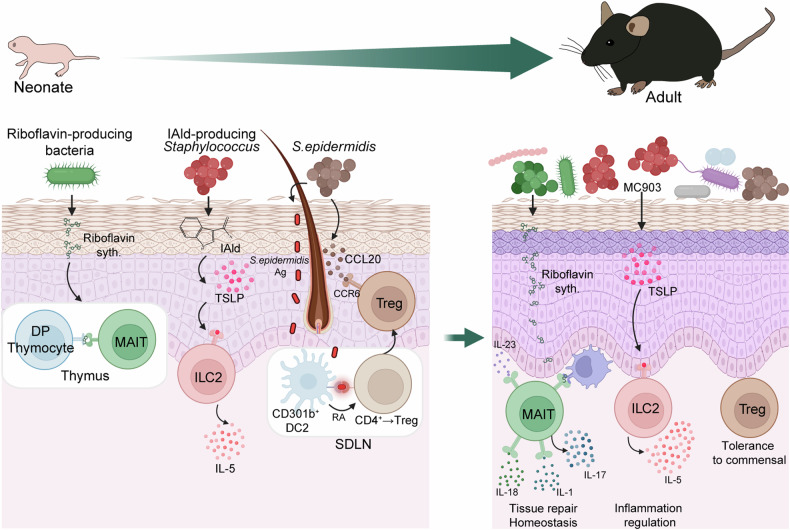
Role of skin microbiota in early life. In early life, exposure to skin microbiota is a critical event for immune cell imprinting. Riboflavin-producing bacteria drive the development of mucosal-associated invariant T (MAIT) cells by synthesizing riboflavin, which leads to MAIT cell imprinting. These imprinted MAIT cells induce IL-1-dependent IL-17A production, promoting tissue repair when skin is damaged and maintaining homeostasis into adulthood. IAld-producing *Staphylococcus* enhances TSLP production in keratinocytes via IAld, and the increased TSLP primes ILC2 cells to produce IL-5 in early life. The primed ILC2 cells regulate skin inflammation in adulthood in response to stimuli, such as MC903. In addition, *S. epidermidis* induces the development and recruitment of commensal-specific T_reg_ cells through the secretion of CCL20 in upper hair follicles and CD301b^+^ DC2, which recognize *S. epidermidis* antigens in early life. These commensal-specific T_reg_ cells foster tolerance to commensals that persist into adulthood.

Mounting evidence reveals that early-life exposure to commensal microbiota affects the host throughout life^66–69^. Similarly, in the skin, early microbial colonization has a lasting impact on the immune system of the host’s skin. Riboflavin-producing commensal microbiota in mouse skin imprint mucosal-associated invariant T (MAIT) cells in early life, supporting maintaining homeostasis and exerting long-lasting effects throughout the host’s lifespan by promoting IL-17A production^22^ (Fig. 2). In addition, early-life exposure of mouse skin to *S. epidermidis* plays a critical role in shaping the adaptive immune system, particularly by promoting the tolerance of T_reg_ cells to the microorganisms through the RALDH expression of CD301b^+^ DCs. This exposure influences the ability to maintain tolerance to bacteria throughout the host’s life^5,6^ (Fig. 2). *Staphylococcus* species, including *S. lentus*, *Staphylococcus xylosus*, *S. epidermidis* and *S. aureus*, produce IAld, a tryptophan metabolite, which augments TSLP in keratinocytes. This process imprints skin ILC2s to produce IL-5 in the skin of neonatal mice. Subsequently, the imprinted ILC2s regulate skin inflammation until adulthood^23^ (Fig. 2). Monocytes infiltrating neonatal mouse skin in response to commensal microbiota regulate the type 17 immune response and contribute to maintaining homeostasis^70^. In addition, early-life antibiotic treatment exacerbated pathology in adulthood in a mouse model of psoriasis^71^. In mice with disrupted epidermal barriers, commensal-specific CD4^+^ T effector cells predominate over T_reg_ cells, and this phenomenon persists from the neonatal stage into adulthood^72^. In humans, skin microbiota during the first year of life is associated with skin barrier integrity and the risk of atopic dermatitis later in life^73^. Furthermore, infants exposed to *S. aureus* before the age of 2 months show a low incidence of atopic dermatitis by their first year^74^. This suggests that early colonization by *S. aureus* may protect against developing atopic dermatitis later in life.

These observations highlight the importance of early-life commensal microbiome colonization and its long-term impact on host health. Identifying skin microbiota and the mechanisms that have lasting effects when encountered in early life may be important for establishing strategies to adjust the composition of the commensal microbiome, promoting improved health outcomes throughout life.

## Commensal skin microbiota and diseases

### Atopic dermatitis

Atopic dermatitis is a representative inflammatory skin disease linked to skin microbiota, which occurs in approximately 20% of children and 10% of adults. Most cases of atopic dermatitis develop between 2 and 6 months of age and either resolve or persist into adulthood. Furthermore, atopic dermatitis is more prevalent in developed countries than in developing countries^75–78^. Atopic dermatitis is caused by either a deficiency of filaggrin (an epidermal structural protein) or environmental factors.

In atopic dermatitis, the epidermal barrier is disrupted. In addition, microbial dysbiosis, characterized by the proliferation of *S. aureus* and *Malassezia* yeasts, is observed in atopic skin (Table 2). Damaged epidermal barriers recruit ILC2s through the secretion of IL-18, TSLP and IL-33, which activate immune cells, including T helper 2 cells (Th2s) and eosinophils, thereby exacerbating inflammation^25,76,79–81^. *S. aureus* is notably prevalent in atopic skin^82–84^. Although it remains unclear whether epidermal barrier disruption or excessive *S. aureus* expansion in the skin is the primary event leading to atopic dermatitis (Table 2), it is evident that, in atopic skin, characterized by elevated pH and reduced antimicrobial peptide expression, *S. aureus* proliferates more effectively and exacerbates inflammation^85–88^. This suggests a relationship between epidermal barrier integrity and microbial expansion, which contributes to the severity of the condition.

**Table 2:** Diseases and microbiota of the skin.

Disease	Alteration of skin microbiota	Description	Reference
Atopic dermatitis	*Staphylococcus* ↑ *Staphylococcus aureus* ↑ *Staphylococcus epidermidis* ↑ *Staphylococcus hominis* ↑ *Cladosporium* ↑ *Leptosphaeria* ↑ *Debaryomyces* ↑ *Cutibacterium acnes* ↑or ↓ Lactobacilli ↓ *Burkholderia* spp. ↓	Atopic dermatitis is a persistent condition characterized by skin inflammation, redness and irritation. It is often triggered by environmental factors or a lack of filaggrin, leading to intense itching and complications such as swelling, crusting and scaling due to scratching.	^132,141,142^
Ichthyosis vulgaris	*Staphylococcus* ↑ Actinobacteria ↑ *Corynebacterium* ↑ *Cutibacterium acnes* ↑	Ichthyosis vulgaris, a nonsyndromic hereditary form of ichthyosis, accounts for >95% of all cases. It is caused by a heterozygous loss-of-function mutation in the filaggrin gene. Typically presenting in infancy, symptoms include xerosis, keratosis pilaris, palmoplantar hyperlinearity, scaly dermatitis and erythroderma.	^132^
Netherton syndrome	*Staphylococcus* ↑ *Actinobacteria* ↑ *Cladosporium* ↑ *Staphylococcus epidermidis* ↑ *Staphylococcus hominis* ↑ *Cutibacterium acnes* ↑ *Corynebacterium tuberculostearicum* ↑ *Malassezia globose* ↑ Proteobacteria ↓ *Enhydrobacter* ↓ Clostridia ↓ *Lactobacillus* ↓	Netherton syndrome is a rare autosomal recessive form of ichthyosis that provokes chronic skin inflammation. It typically presents with a triad of symptoms: congenital ichthyosiform erythroderma, trichorrhexis invaginata and atopic diathesis. The condition results from pathogenic mutations in the *SPINK5* gene.	^132^
Psoriasis	*Corynebacterium. simulans* ↑ *Corynebacterium. kroppenstedtii* ↑ *Finegoldia* ↑ *Neisseriaceae* ↑ *Malassezia restricta* ↑ *Cutibacterium acnes* ↓ *Lactobacilli spp* ↓ *Lactobacillus iners* ↓ *Burkholderia spp* ↓	Psoriasis is a noninfectious autoimmune disorder marked by characteristic patches of abnormal skin, which are typically dry, itchy and scaly and appear red, pink or purple. As revealed by immunological and genetic studies, IL-17 and IL-23 have been identified as the primary drivers of psoriasis development.	^142,143^
Hidradenitis suppurativa	*Porphyromonas* ↑ *Peptoniphilus* ↑ *Propionibacterium* ↓ *Cutibacterium acnes* ↓ *Corynebacterium striatum* ↓ *Staphylococcus epidermidis* ↓, *Micrococcus luteus* ↓ *Kocuria* ↓	Hidradenitis suppurativa is a persistent inflammatory disorder affecting hair follicles, marked by recurrent nodules, abscesses and prolonged suppurative lesions, commonly occurring in the axillary, inguinal and perineal regions.	^144,145^
Acne vulgaris	Firmicutes ↑ *Staphylococcus* ↑ *Enterococcus* ↑ Proteobacteria ↓ *Acinetobacter* ↓ Lachnospiraceae ↑ Clostridiales ↑ Moraxellaceae ↑ *Prevotella* ↑ *Lactococcus garvieae* ↑ *Achromobacter* ↓ *Stenotrophomonas* ↓ *Porphyromonas* ↓ *Prevotella* ↓ *Pseudomonas* ↓ *Propionibacterium* ↓	Acne vulgaris is a chronic inflammatory condition affecting the pilosebaceous units, primarily driven by increased sebum production induced by androgens, altered keratinization, inflammation and the proliferation of lipophilic anaerobic bacteria. Common symptoms include crusting of skin bumps, cysts, papules, pustules and erythema surrounding lesions, as well as various forms of scarring such as white- and blackheads.	^146,147^

In humans, early exposure to *S. aureus* before 2 months of age is associated with a reduced incidence of atopic dermatitis by the age of 1 year (ref. ^74^). This finding suggests a concept similar to the hygiene hypothesis. In addition, it is known that certain bacterial species exhibit diverse strains within individuals, and transmission of skin microbiota occurs between individuals. Furthermore, genetic mixing within microbiota can regulate virulence factor expression in bacteria^89^. Therefore, *S. aureus* colonized before 2 months of age might differ in virulence factor expression compared with *S. aureus* colonizing the skin of adults with atopic dermatitis. This difference could be attributed to variations in skin microbiota composition between infants and adults. However, the precise mechanism underlying this effect is not yet well understood and requires further investigation.

Various types of mouse models for atopic dermatitis are available. Among them, filaggrin mutant mice develop spontaneous atopic-like skin inflammation, similar to that in humans, and show an increased abundance of *Staphylococcus* in the skin microbiota^39^. Furthermore, the 2,4-dinitrochlorobenzene-induced atopic dermatitis model shows an increased *Staphylococcus* population in the skin microbiota composition through mast cell activation^90^.

Therefore, atopic dermatitis, epidermal disruption, excessive *S. aureus* expansion and commensal skin microbiota dysbiosis are closely associated^91^ (Table 2). Investigating the relationship among the epidermal barrier, skin microbiota and immune cells supports the development of strategies for treating skin diseases.

### Psoriasis

Psoriasis is a chronic inflammatory skin disease characterized by epidermal hyperplasia and parakeratosis^92^. It affects approximately 1–3% of the global population^93^. The onset of psoriasis is driven by genetic predisposition as well as environmental factors, including streptococcal throat infections, alcohol consumption, smoking, lithium exposure and obesity^94^.

The IL-23–IL-17 axis is a key driver of chronic inflammation and keratinocyte proliferation in psoriasis, promoting the activation of T cells, DCs and keratinocytes^94^. Similarly, this axis is central to the imiquimod (IMQ)-induced mouse model of psoriasis^95^.

Furthermore, the pathogenesis of psoriasis is closely linked to the dysbiosis of skin microbiota (Table 2). In the IMQ-induced model, conventional mice exhibit more severe psoriatic inflammation than germ-free or antibiotic-treated mice^96^. In addition, *S. lentus* and Proteobacteria populations increase in the skin of IMQ-induced model mice^97,98^. *Staphylococcus warneri* and *C. albicans* exacerbate psoriasis pathogenesis in the IMQ-induced model^99,100^. By contrast, *Staphylococcus cohnii* plays a protective role in IMQ-induced psoriasis^45^.

Although the correlation between psoriasis and skin microbiota is weaker than that observed with atopic dermatitis, antibiotic treatment in early life has been shown to exacerbate psoriasis pathology in adulthood in mouse models^71^. In addition, considering the impact of various skin-microbiota-derived metabolites on psoriasis, microbiota may play a critical role in psoriasis pathogenesis^38,101^.

### Acne vulgaris

Acne vulgaris (acne) is an inflammatory skin disease of the pilosebaceous unit. Acne affects approximately 15–20% of individuals aged 15–17 years with moderate-to-severe symptoms. Various environmental factors, including ultraviolet exposure, dietary factors, smoking, stress and lifestyle, contribute to the development of acne^102^. Furthermore, inflammatory signaling via CD4^+^ lymphocytes, keratinization of pilosebaceous ducts and increased sebum production are involved in acne development^103,104^. However, other factors involved in acne development remain unclear.

*C. acnes* has long been proposed to be associated with acne, but several studies suggest that it does not exhibit overgrowth in acne lesions or the skin of patients with acne relative to that of healthy controls. In fact, *C. acnes* populations in patients with acne and healthy controls are similar^103,104^. Nonetheless, reduced microbial diversity and *C. acnes* dysbiosis may trigger acne development (Table 2). The interaction between *C. acnes* and other commensal microorganisms, such as *S. epidermidis*, is crucial for maintaining skin homeostasis and can influence acne progression^103,104^. Furthermore, skin microbiota composition differs according to the severity of acne^105^. These findings suggest that acne may develop from the complex interplay between skin microbiota and the immune system.

### Commensals and pathogens

How do hosts distinguish between commensals and pathogens? When the balance of various skin commensals is maintained, they do not exhibit pathogenic characteristics, allowing the host to remain tolerant to them. Opportunistic pathogens originate from microorganisms, including commensals, and typically do not infect healthy hosts. However, they can cause infections when the host’s immune system is compromised or dysbiosis occurs. Notably, in some cases, the overgrowth of specific commensals or their production of danger signals can further exacerbate this imbalance, triggering pathogenic responses in the host^106–113^.

In neonatal skin, colonization by the commensal microorganism *S. epidermidis* induces the formation of *S. epidermidis*-specific T_reg_ cells, promoting tolerance. By contrast, when pathogenic *S. aureus* colonizes the skin, its secretion of α-toxin limits T_reg_ cell formation and promotes the differentiation of T effector cells, preventing the establishment of tolerance. This demonstrates that the host has a system for distinguishing between commensals and pathogens^59,114^.

Although *S. epidermidis* inhibits the growth of *S. aureus* as a commensal, it can become pathogenic through secretion of potent virulence factors such as proteases, lipases and phenol-soluble modulins and cause infections under certain conditions^111,115,116^. *C. acnes* is among the skin commensals that produce propionic acid, facilitating the maintenance of appropriate skin pH and inhibiting the growth of pathogens^117^. However, *C. acnes* also plays a pathogenic role in acne lesions, contributing to the development of acne vulgaris^118^.

Furthermore, *Malassezia*^109^ and *C. albicans*^119^ reside on human skin as commensals under steady-state conditions. However, when skin barrier defects occur, skin immune responses are compromised or the skin is exposed to antibiotics, these organisms can act as pathogens. Moreover, much research on the diverse and specific mechanisms by which the host recognizes commensal microorganisms and pathogens remains to be conducted. Understanding these mechanisms could lead to more effective treatments for various skin infections.

## Conclusion and perspectives

Commensal skin microbiota influence multiple host skin functions. This Review discusses how various commensal skin microbiota impact the host during early life and beyond.

Commensal microbiota engage in diverse interactions, including physical, environmental and chemical mechanisms, as well as biofilm formation and competition^120,121^. Among fungi, *C. albicans* interacts with various bacterial species in multiple organs, such as the gut, lungs, vagina and oral cavity^120,122^.

In the oral cavity and gut, commensal bacteria, including *Fusobacterium nucleatum*, *Lactobacillus acidophilus*, *Lactobacillus reuteri*, *Lactobacillus casei GG* and *Bifidobacterium animalis*, inhibit *C. albicans* hyphal formation and biofilm development^122^. In addition, certain bacteria suppress the yeast-to-hypha transition in *C. albicans*^122^. Conversely, interactions between *C. albicans* and commensal bacteria, such as *Streptococcus* species, can exacerbate infections and inflammation in the oral cavity and gut^122^.

The skin harbors a complex microbiota, encompassing multiple kingdoms, including fungi such as *C. albicans*, *Malassezia* and *Aspergillus*^122,123^.The composition of skin fungi undergoes dynamic changes during life stages, including early life, puberty and adulthood. *Malassezia* has been shown to inhibit biofilm formation by *S. aureus* on the skin^124^ (Fig. 1). Despite the presence of fungi on the skin and the skin immune system’s responses to these organisms^125^, evidence regarding fungi–bacteria interactions on the skin remains limited compared with other organs such as the oral cavity and gut. Further investigation into these interactions could enhance our understanding of the skin microbiota and elucidate the complex crosstalk between fungi, bacteria and the host immune system.

In early life, various skin commensals have a long-lasting impact on the host. Although it is known that these commensals imprint specific cell types, the precise mechanisms by which they alter lifelong cellular activity remain unclear.

Epigenetic mechanisms are known to regulate genomic imprinting^126^. Many environmental factors can affect gene expression, leading to epigenetic changes^127^, and microbiota influence the host’s epigenetic programming. In the mouse colon, microbiota-induced changes in the degree of hypermethylation of specific genes have been observed^128^. In addition, microbial metabolites, such as short-chain fatty acids and proteins, impact epigenetic changes in the intestinal epithelium^129,130^. In the mouse skin, *S. epidermidis* elevated chromatin accessibility at type 2 immune gene loci in T cytotoxic17 cells^10^. Furthermore, butyric acid produced by *S. epidermidis* inhibits *S. aureus* growth by acting as a histone deacetylase inhibitor^131^.

In patients with atopic dermatitis, genetic deficiencies play a role, but environmental factors also notably contribute to disease pathogenesis. Moreover, the prevalence of atopic dermatitis is higher in developed than in developing countries. The environmental differences between these regions lead to variations in skin microbiota populations, which, in turn, might influence disease prevalence. Thus, numerous studies have investigated the relationship between atopic dermatitis and epigenetic changes. DNA methylation, histone modification and noncoding RNA have been found to differ between the skin of patients with atopic dermatitis and healthy individuals^131^.

Therefore, skin microbiota influence the epigenetic changes of the skin immune system, which can be transient or permanent. Thus, permanent immune cell imprinting by commensal skin microbiota in early life may be attributed to microbiota-induced epigenetic changes. Germ-free mice and techniques, such as ChIP-seq, ATAC-seq and scATAC-seq, could be used to investigate the epigenetic alterations in keratinocytes and skin immune cells induced by microbiota in early life.

Understanding the interactions among microbiota and epithelial and immune cells in the skin, along with their epigenetic modifications, could facilitate the identification of precise targets and offer effective solutions for skin diseases resulting from epidermal barrier disruption and dysbiosis.
